# Supplementary material for: Synthesis of Spin-Labelled Bergamottin: A Potent CYP3A4 Inhibitor with Antiproliferative Activity
Source: Int J Mol Sci. 2020 Jan 13;21(2):508. doi: 10.3390/ijms21020508 (PMC7013880; doi:10.3390/ijms21020508)
Supplement: Supplementary file 1 [file ijms-21-00508-s001.pdf]

NMR spectra of new compounds

α-methyl carbons are usually invisible in ^{13}C NMR spectra in the case of piperidin type of nitroxides

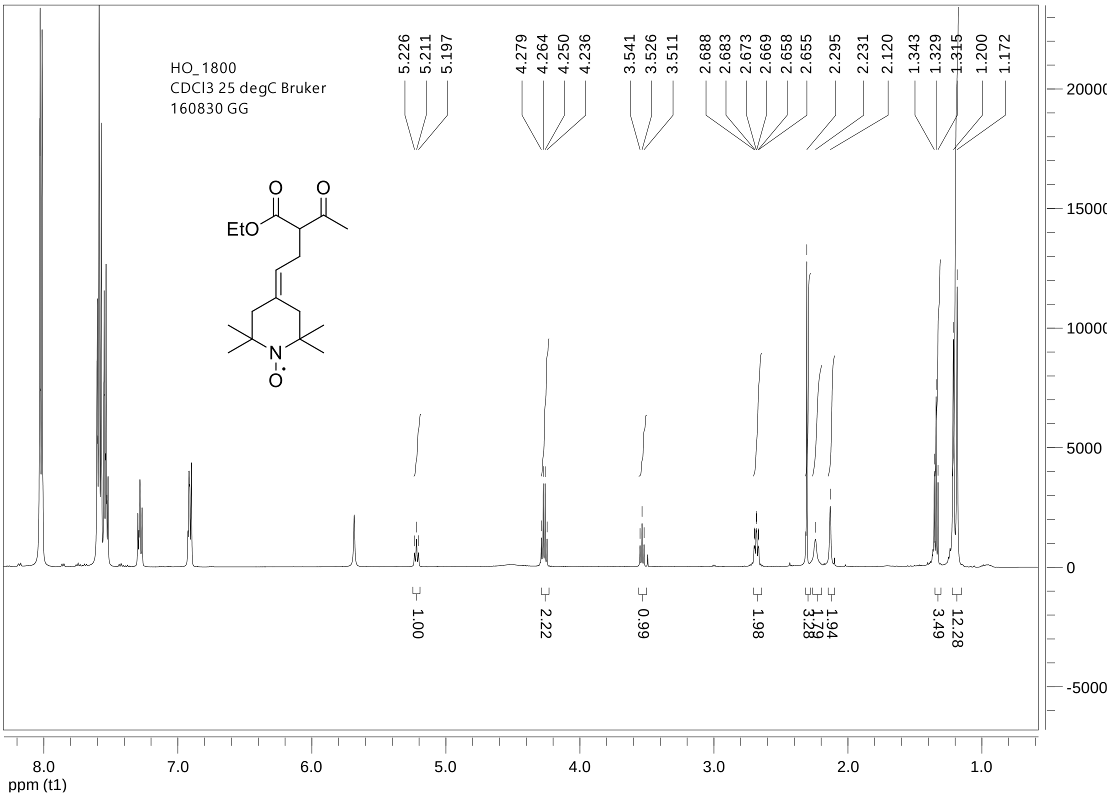

compound **4**

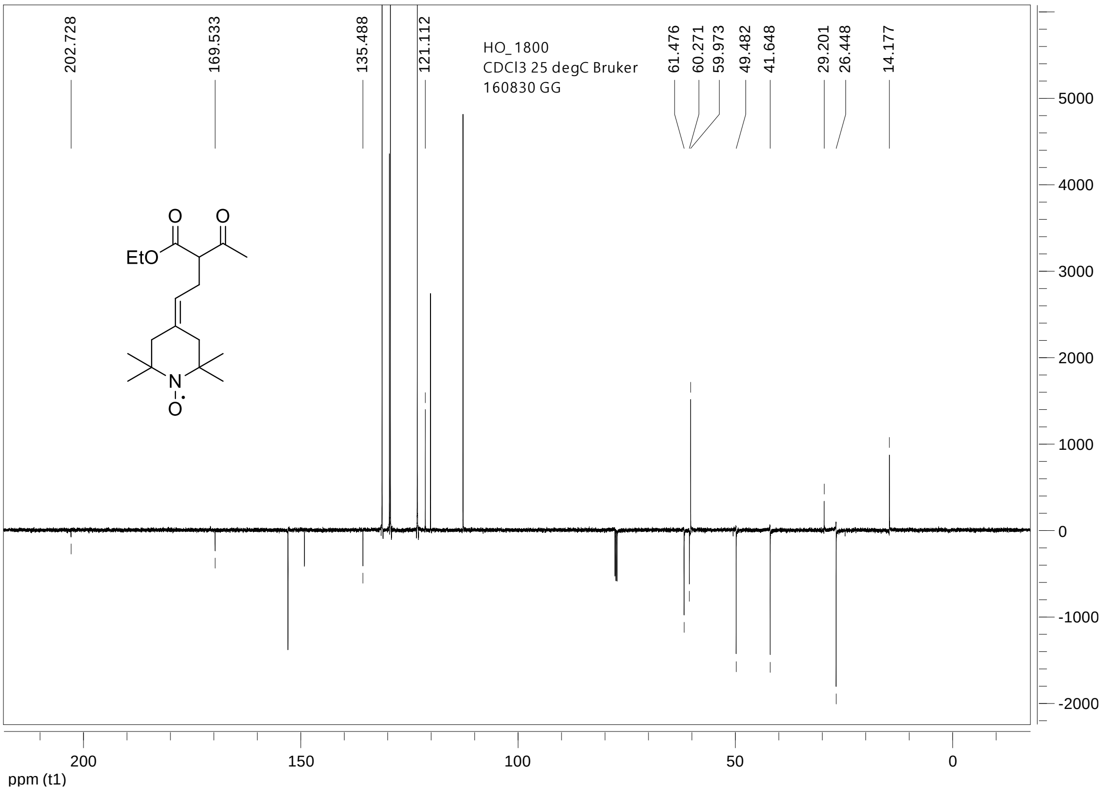

compound **4**

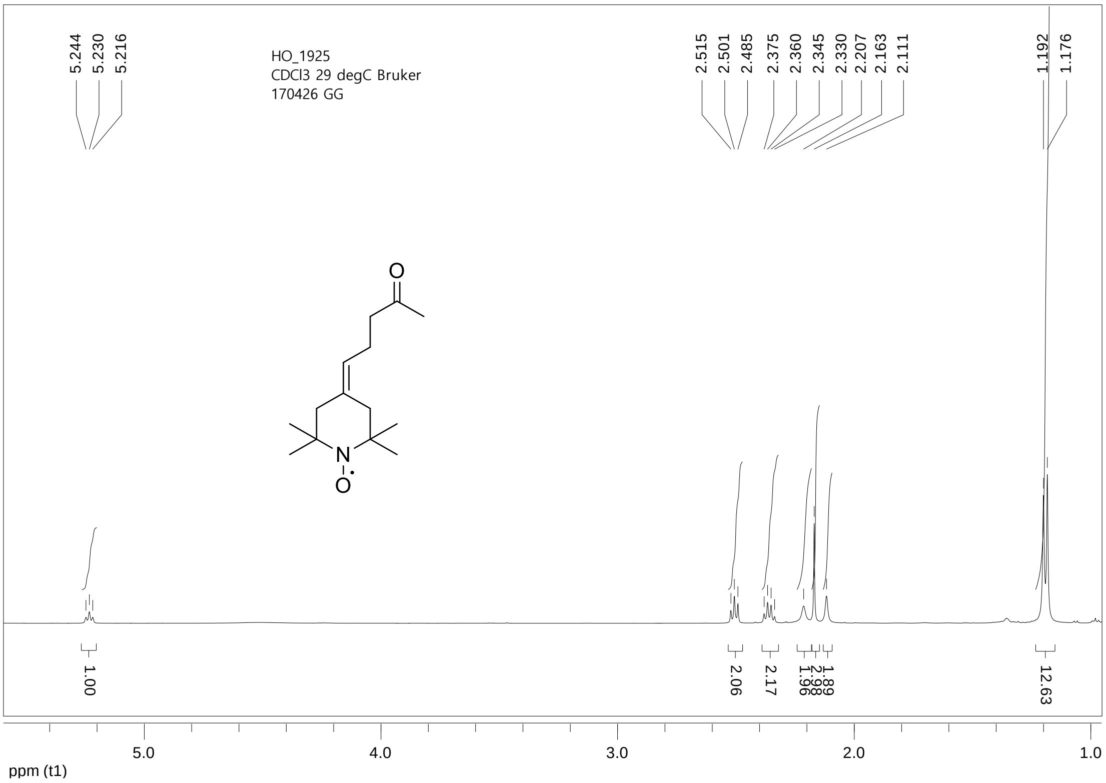

compound **5**

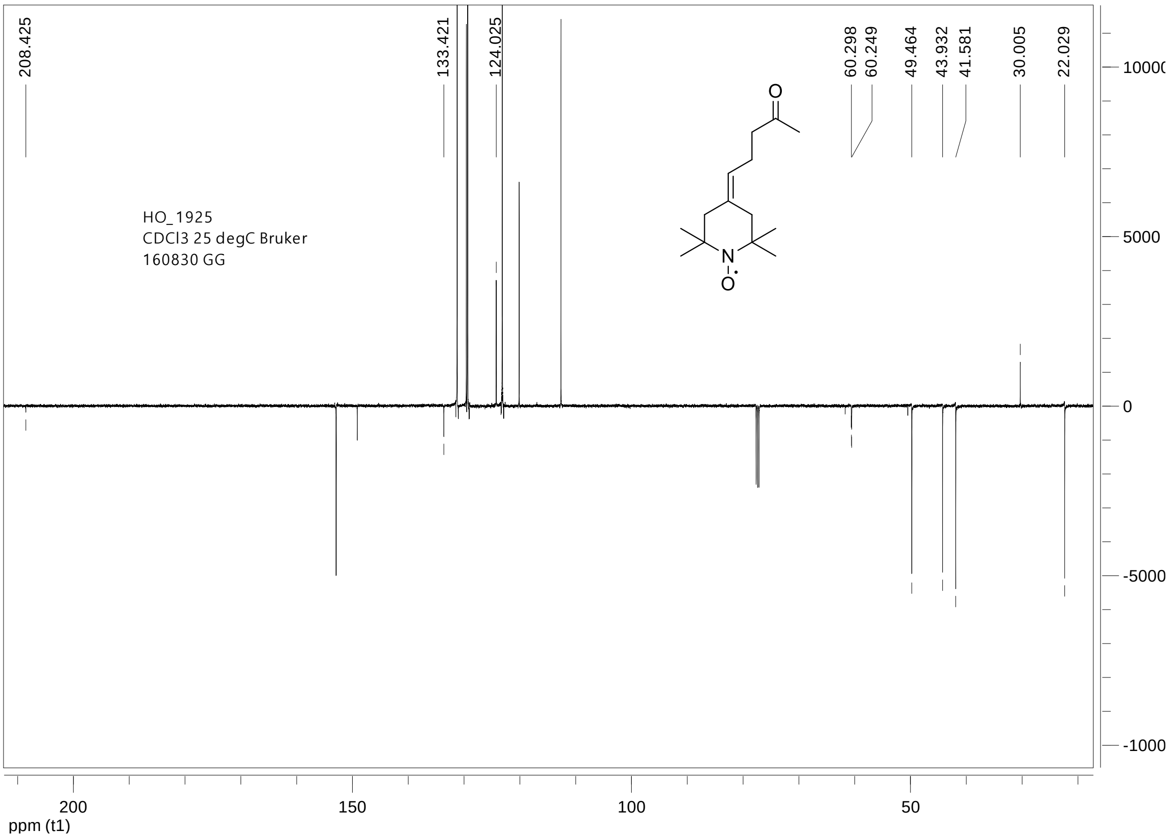

compound **5**

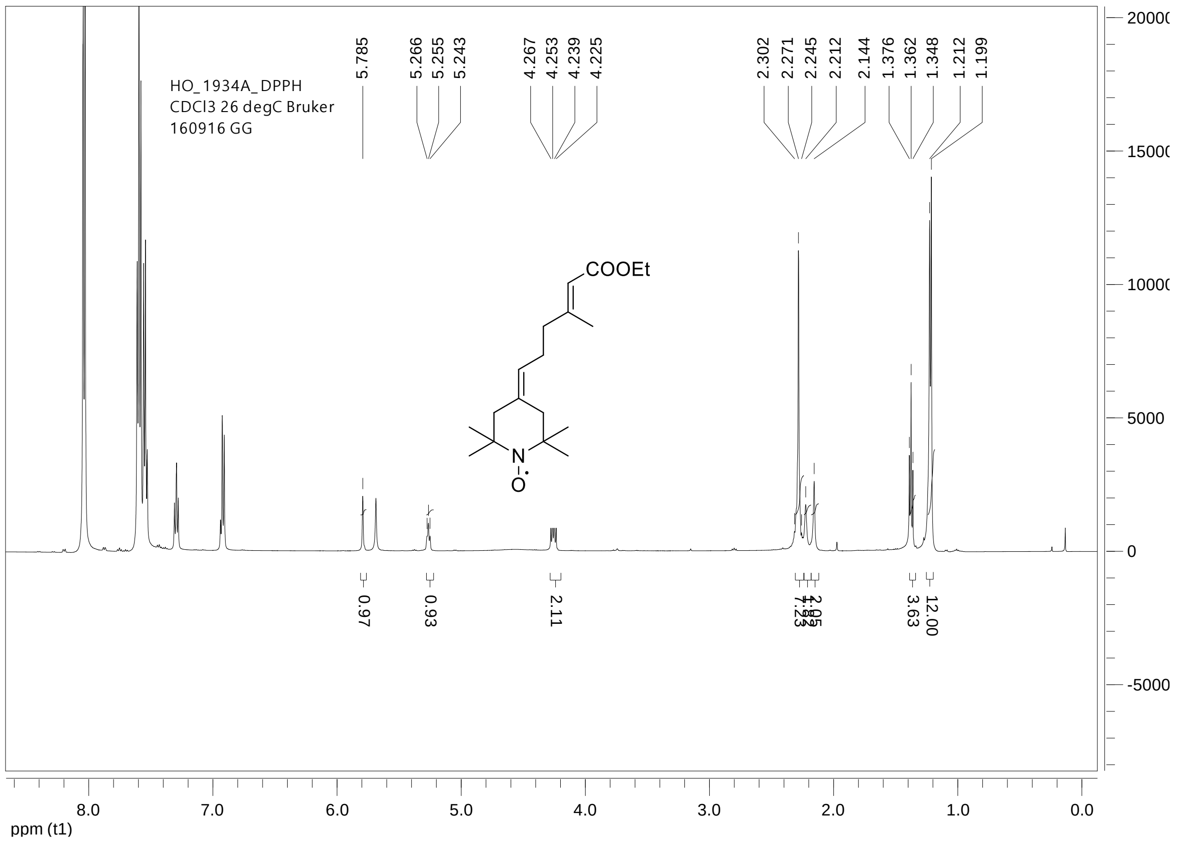

compound **6**

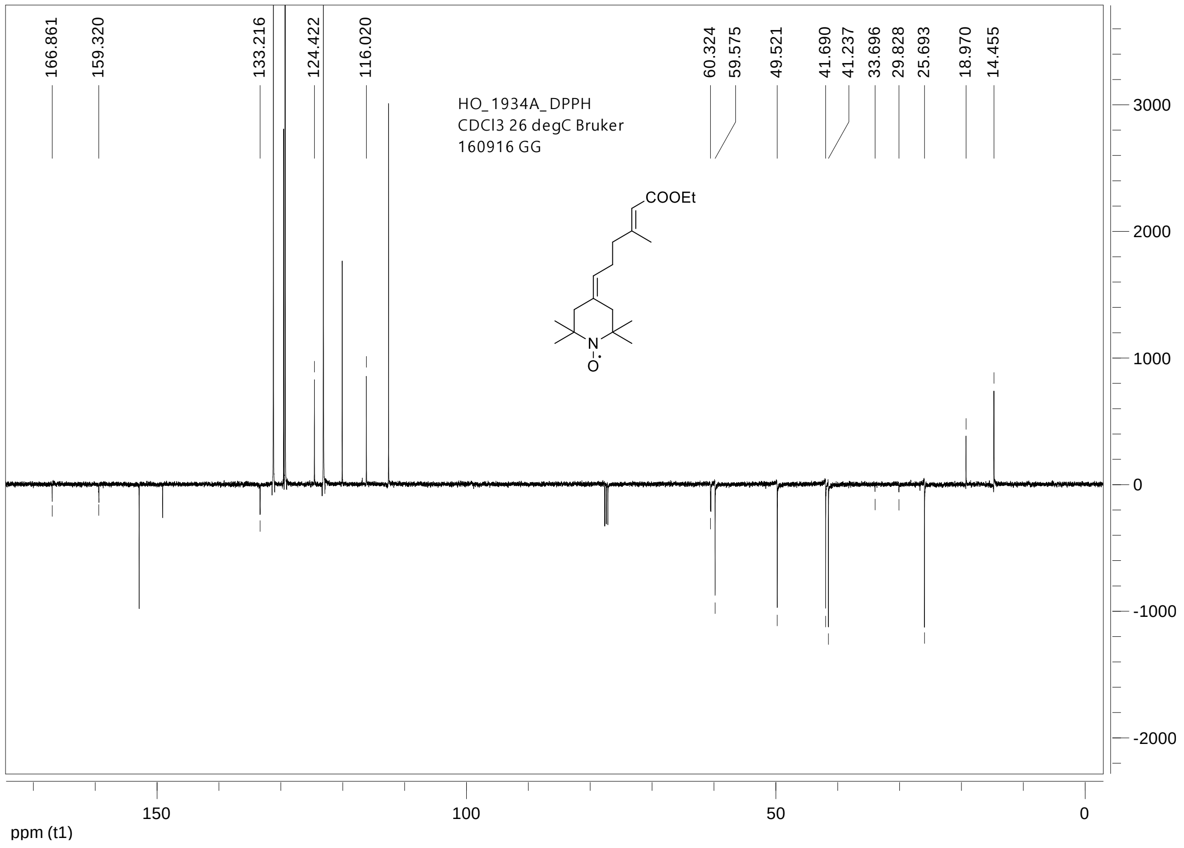

compound **6**

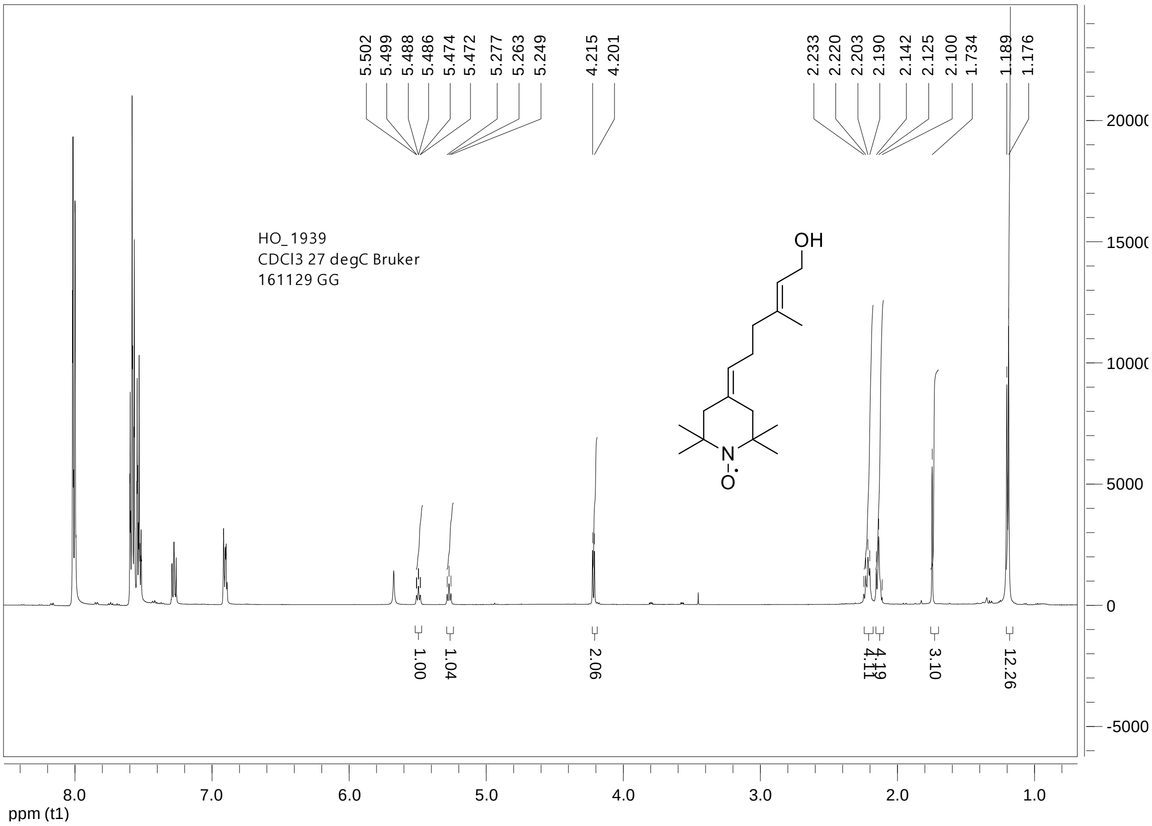

compound **7**

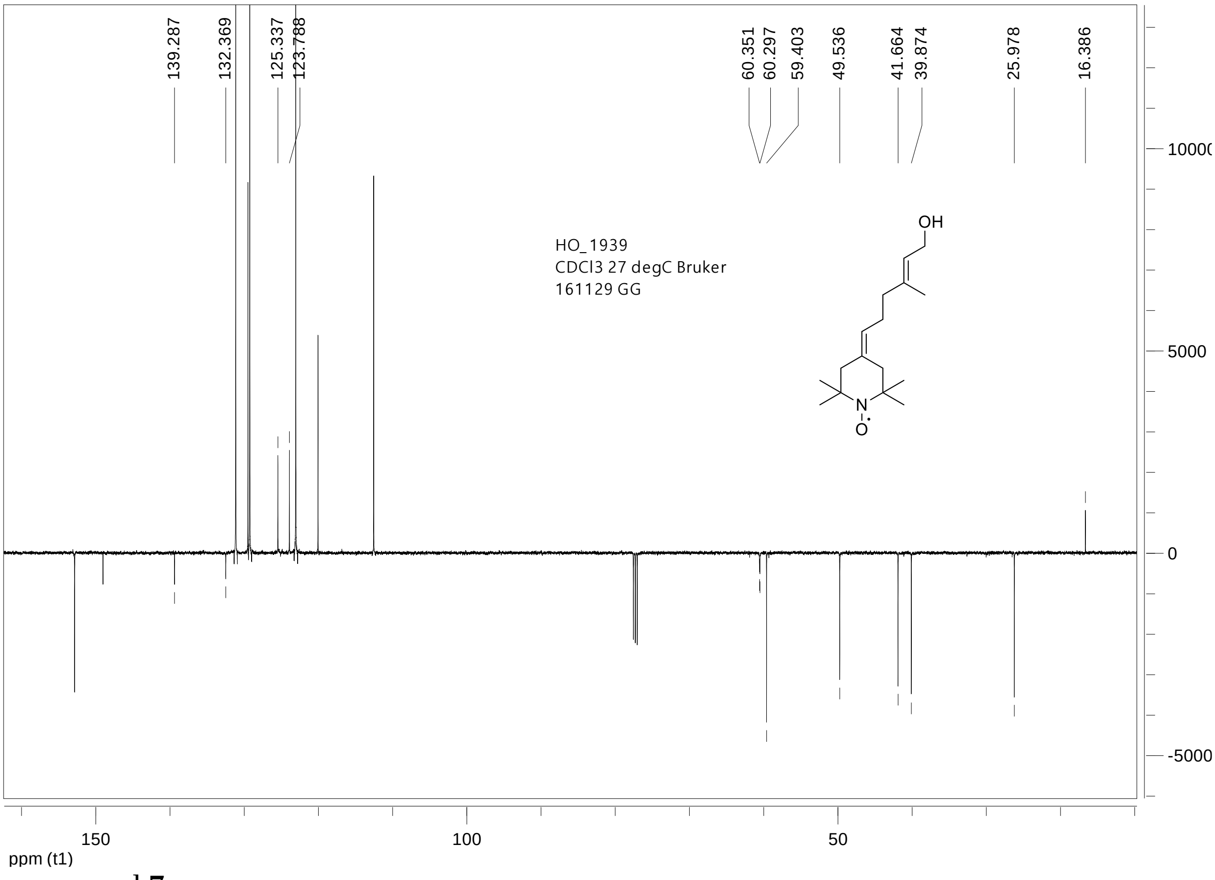

compound **7**

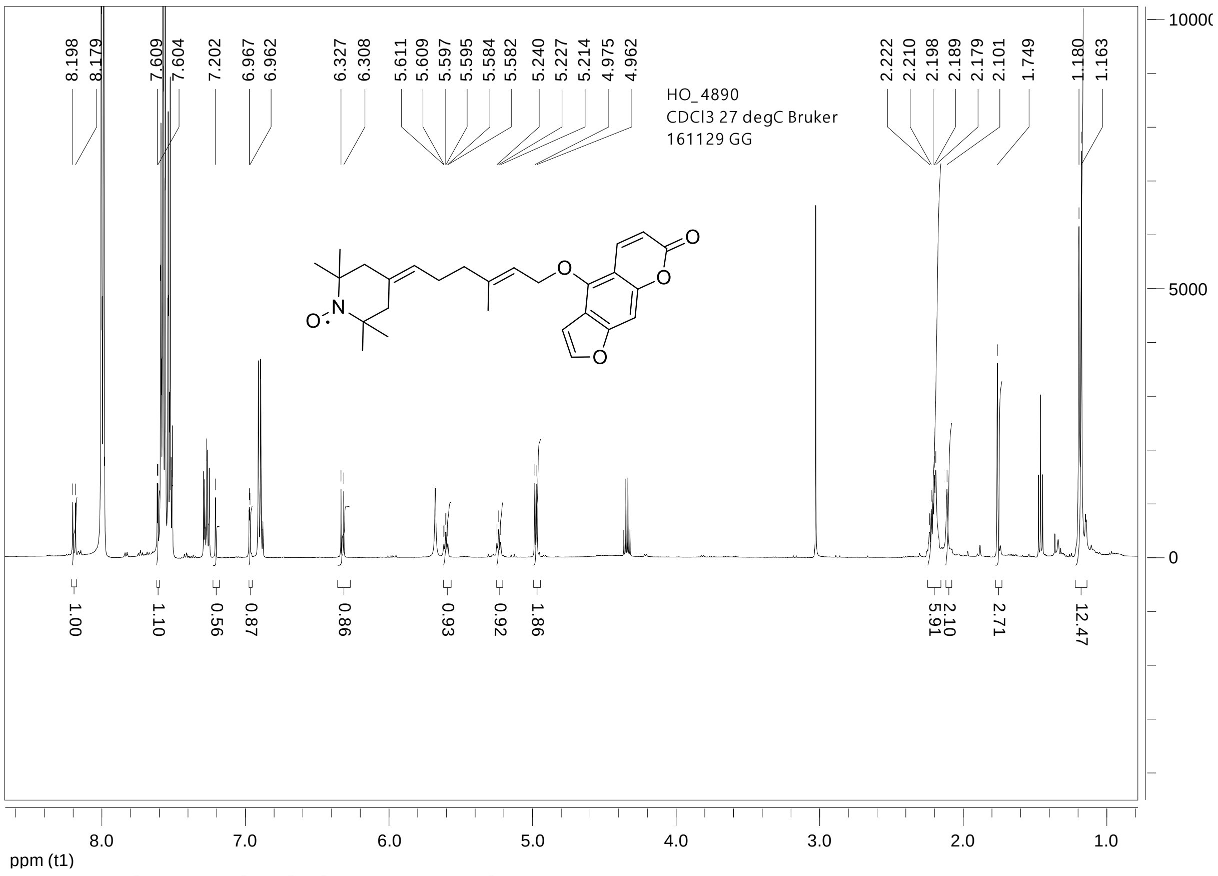

compound **10** (with ethyl acetate residues)

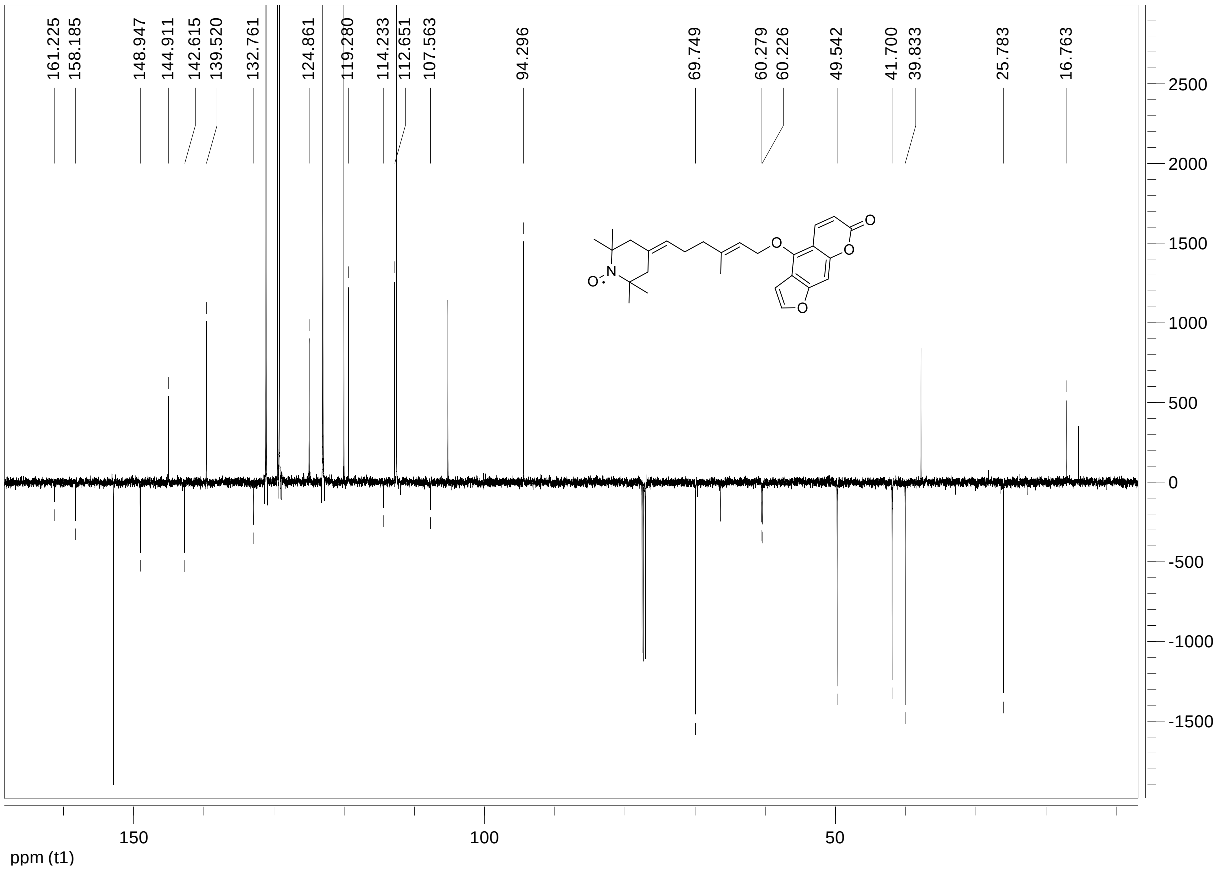

compound **10** (with ethyl acetate residues)
